# Superiorization of projection algorithms for linearly constrained inverse radiotherapy treatment planning

**DOI:** 10.3389/fonc.2023.1238824

**Published:** 2023-10-26

**Authors:** Florian Barkmann, Yair Censor, Niklas Wahl

**Affiliations:** ^1^ Institute for Machine Learning, Department of Computer Science, ETH Zürich, Zurich, Switzerland; ^2^ Department of Medical Physics in Radiation Oncology, German Cancer Research Center (DKFZ), Heidelberg, Baden-Württemberg, Germany; ^3^ Heidelberg Institute for Radiation Oncology (HIRO) and National Center for Radiation Research in Oncology (NCRO), Heidelberg, Baden-Württemberg, Germany; ^4^ Department of Mathematics, Faculty of Natural Sciences, University of Haifa, Haifa, Israel

**Keywords:** radiation therapy treatment planning, inverse planning, constrained treatment plan optimization, IMRT, superiorization method, feasibility-seeking algorithm

## Abstract

**Objective:**

We apply the superiorization methodology to the constrained intensity-modulated radiation therapy (IMRT) treatment planning problem. Superiorization combines a feasibility-seeking projection algorithm with objective function reduction: The underlying projection algorithm is perturbed with gradient descent steps to steer the algorithm towards a solution with a lower objective function value compared to one obtained solely through feasibility-seeking.

**Approach:**

Within the open-source inverse planning toolkit matRad, we implement a prototypical algorithmic framework for superiorization using the well-established Agmon, Motzkin, and Schoenberg (AMS) feasibility-seeking projection algorithm and common nonlinear dose optimization objective functions. Based on this prototype, we apply superiorization to intensity-modulated radiation therapy treatment planning and compare it with (i) bare feasibility-seeking (i.e., without any objective function) and (ii) nonlinear constrained optimization using first-order derivatives. For these comparisons, we use the TG119 water phantom, the head-and-neck and the prostate patient of the CORT dataset.

**Main results:**

Bare feasibility-seeking with AMS confirms previous studies, showing it can find solutions that are nearly equivalent to those found by the established piece-wise least-squares optimization approach. The superiorization prototype solved the linearly constrained planning problem with similar dosimetric performance to that of a general-purpose nonlinear constrained optimizer while showing smooth convergence in both constraint proximity and objective function reduction.

**Significance:**

Superiorization is a useful alternative to constrained optimization in radiotherapy inverse treatment planning. Future extensions with other approaches to feasibility-seeking, e.g., with dose-volume constraints and more sophisticated perturbations, may unlock its full potential for high performant inverse treatment planning.

## 1 Introduction

Numerical optimization methods lie at the heart of state-of-the-art inverse treatment planning for intensity-modulated radiation therapy (IMRT) (1). Usually, a clinical prescription of the treatment goals forms the input to a nonlinear multi-criteria optimization (MCO) problem with or without additional constraints, depending on the desired patient dose distribution.

During the translation of the clinical goals into an MCO problem, one distinguishes between objectives, i.e., soft goals that compete with each other, and hard constraints designed to ensure, for example, maximal tolerance doses in an organ-at-risk (OAR) and minimal dosage of the target. This versatile approach enables the treatment planner to employ arbitrary combinations of suitable (convex) nonlinear objective functions along with any choice of constraints on the voxels’ doses.

This mathematical modeling allows numerical optimization of the fluence of beam elements (beamlets) using a pre-computed normalized dose mapping (2). The resulting constrained nonlinear optimization problem is frequently solved by applying an extended (quasi-)Newton approach with sequential quadratic programming (SQP) and/or interior-point methods (1–7). Until now, the capabilities of inverse planning have been substantially extended through multi-criteria Pareto optimization with subsequent exploration of the Pareto surface (8, 9) or stochastic/robust optimization (10).

Computational difficulties may arise in the constrained nonlinear optimization approach. First, optimal convergence for problems of typical size in radiotherapy is tied to the availability of computationally efficient second-order derivatives. While, for example, van Haveren and Breedveld (11) showed that for many typical functions efficient formulations can be found, current research persistently adds new quantities, optimization strategies, and new types of problem formulations to inverse planning for photons and particles (see, e.g., 12–18) to which such strategies might not be directly applicable. Second, a common approach among successful optimizers for nonlinear constrained optimization is to transform the constrained problem into an unconstrained problem using, for example, barrier functions (in the case of interior point methods, e.g., 3, 19) and the method of Lagrange multipliers in combination with slack variables (3, 19, 20). This creates a computational burden when the number of constraints increases. Handling many constraints as, for example, linear inequalities for many or all individual voxel dose bounds, can inflate the computational effort because each constraint requires a Lagrange multiplier and an additional slack variable. Possible “workarounds” include minimax-optimization in combination with auxiliary variables or usage of continuous and differentiable maximum approximations like the LogSumExp and softmax functions (5).

Taking a step back, however, to the starting days of treatment planning research, shows that one does not necessarily need to use a mathematical *optimization* approach to solve the purely linearly constrained IMRT problem but could use feasibility-seeking projection algorithms instead (21, 22).

In the context of IMRT, such bare feasibility-seeking translates to seeking a feasible solution that will obey the prescribed lower and upper dose bounds on doses in voxels, without aiming to optimize any objective function. While, in general, a bare feasibility-seeking task can be translated to a constrained optimization problem with a zero objective function, the literature demonstrates a wide spectrum of many efficient feasibility-seeking algorithms not derived from translation of the bare feasibility-seeking task to a constrained optimization problem (see, e.g., 23). If no feasible solution is found, these algorithms find a proximal solution, similar to the piece-wise least-squares approach. Even though they have seen further development over the last decades (24) and, more recently, also extension to dose-volume constraints (25–27), numerical optimizers have been the preferred choice in the field due to their abilities to handle the nonlinear objective functions, e.g., (generalized) equivalent uniform dose (EUD), which are often desired when prescribing treatment goals.

The work presented here now combines nonlinear objective functions as used in optimization with feasibility-seeking within linearly constraining dose bounds by applying the superiorization method (SM). To do so, the SM uses a *superiorized version of the basic algorithm*, the latter being a user-chosen iterative feasibility-seeking algorithm, which is perturbed by interlacing reduction steps of the chosen (nonlinear) objective function. This practically steers the iterates of the feasibility-seeking algorithm to a feasible solution point with a “superior”, i.e., smaller or equal objective function value, which is not necessarily a constrained minimization point.

The superiorization method thus works with the constraints data and the user’s choice of objective function, much alike constrained optimization methods would. But it does not aim at an optimal point that minimizes the objective over all constraints like the latter do. In contrast, the SM aims at a point that will fulfill all constraints and have a reduced – not necessarily minimal – objective function value. Not finding the optimal solution, but instead aiming for a satisfactory or adequate result, is a reasonable decision strategy (“Satisficing”, see 28), particularly considering the degeneracy of the IMRT optimization problem (29). Hence, this aim suffices for the purpose of generating acceptable treatment plans. Combined with the simplicity of the gradient descent steps (i.e., not relying on second-order derivatives), superiorization can find a solution, in general, faster and with less investment of computing resources, and fewer conditions concerning design of the objective function.

Application of the SM to treatment planning is encouraged by the flexibility it has shown for applications in multiple fields:^1^ It has demonstrated its effectiveness for image reconstruction in single-energy computed tomography (CT) (31, 32), dual-energy CT (33) and, more recently, in proton CT (34, 35), by reducing total variation (TV) during image reconstruction. The SM has also been successfully applied to diverse other fields of applications, such as tomographic imaging spectrometry (36) or signal recovery (37).

This work is – to the best of our knowledge – the first in-depth investigation of the SM as a potential alternative to constrained minimization algorithms for inverse radiotherapy treatment planning using common objective functions. To date, we could only identify an initial study of the applicability of SM in IMRT utilizing TV as objective function (38), which does not represent common choices in objective function design for treatment planning. Another work considering the use of SM in IMRT used superiorization to boost a specific lexicographic planning approach (39).

Expanding on those preliminary works, we develop, tune, and evaluate a prototypical superiorization solver for radiotherapy treatment planning problems. To show how this SM solver is able to replace a constrained minimization approach, and to maximize reproducibility and re-usability of our work, our superiorization approach is implemented into the validated open source radiation therapy treatment planning toolkit matRad (5) together with an instructive set of scripts to execute and reproduce the results of this work (see section 2.5). Within matRad and its included phantoms and patient cases, the SM is evaluated and tested on full-fledged IMRT and intensity-modulated proton therapy (IMPT) treatment planning problems. We compare to using non-linear constrained optimization using only first-order derivatives like the SM, that is, a quasi-Newton method construction a Hessian approximation.

This paper is structured as follows: In section 2, we describe the approaches and present the specific version of the SM that we use along with the feasibility-seeking algorithm embedded in it. Section 3 includes our computational results. Finally, in section 4, we discuss the potential of SM with possible future developments and conclude our work in section 5.

## 2 Materials and methods

This work compares three approaches to model the treatment planning problem in IMRT: (i) a *nonlinear constrained minimization approach* of minimizing an objective function subject to constraints with a quasi-Newton method relying on first-order derivatives, (ii) the *feasibility-seeking approach* searching for a feasible solution adhering to constraints without considering any objective functions to minimize, and finally, (iii) the *superiorization approach*, which perturbs the feasibility-seeking algorithm to reduce (not necessarily minimize) an objective function by gradient descent steps. Before introducing these approaches, we briefly recap the discretization of the inverse treatment planning problem.

### 2.1 Discretization of the inverse treatment planning problem

Computerized inverse treatment planning usually relies on a spatial discretization of the particle fluence, the patient anatomy, and, consequently, the radiation dose.

The patient is represented by a three-dimensional voxelized grid (image) with n voxels numbered i=1,2,…,n . Based on this image, Q volumes of interest (VOIs) $S_{q},\;q=1,2,\ldots ,Q$ are segmented. This allows us to represent the dose as a vector $\text{d}={\left(d_{i}\right)}_{i=1}^{n}$ , whose i -th component is the radiation dose deposited within the i -th voxel. For each of the segmentations $S_{q}$ , we can then easily identify its dosage by finding $d_{i}$ for all $i\in S_{q}$ .

The radiation fluence is represented as a vector intensities $\text{x}={\left(x_{j}\right)}_{j=1}^{m}$ , whose j -th component is the intensity of the j -th beamlet. The dose deposition $a_{i}^{j}$ for a unit intensity of beamlet j to voxel i can then be precomputed and stored in the *dose influence matrix* $A={\left(a_{i}^{j}\right)}_{i=1,j=1}^{n,m}$ , mapping x to d via d=Ax .

### 2.2 The constrained minimization approach

In the optimization approach to IMRT treatment planning, the clinically prescribed aims are represented by various (commonly differentiable) objective functions which map the vector of beamlet intensities to the positive real numbers (2).

For our purposes, we limit ourselves to objective functions $f_{p}:{\mathbb{R}}^{n}\to \left[0,\infty \right),\;p=1,2,\ldots ,P$ , operating on the radiation dose d as surrogates for clinical, dose-based goals.

A comprehensive, exemplary list of such common objective functions can be found in Wieser et al. (5, **Table 1**) and, for the reader’s convenience, also in **Supplementary Data Sheet** below. These objective functions, which depend on the dose, are related to the intensities x via d=Ax , which is computed at each iterate/change of x during optimization.

**Table 1:** Dose inequalities/prescriptions and penalty weights used for minimization and for AMS feasibility-seeking.

VOI	*w_p_*	tolerance/inequality constraint
Target	1000	59 Gy < ***d <*** 61 Gy
Core	100	***d *** < 20 Gy
Body	30	***d *** < 30 Gy

Wishing to fulfill or decide between multiple clinical goals, the resulting multi-objective optimization problem may be scalarized using a weighted sum of several different individual objective functions for the various VOIs *S_q_*. This approach, first introduced for least-squares (as introduced by 40), can today explore a plethora of objective functions (2, 5) while also satisfying hard constraints (3, 5):


(1) $$\begin{array}{lll} {\text{x}}^{*}= & \underset{x}{\arg\;\min}\sum_{p=1}^{P}w_{p}f_{p}\left(\text{d}\left(\text{x}\right)\right)\\ \text{such}\;\text{that} & c_{t}^{L}\leq c_{t}\left(\text{d}\left(\text{x}\right)\right)\leq c_{t}^{U}, & t=1,2,\ldots ,T,\\ \text{x}\geq 0\;. \end{array}$$

Here $w_{p}\geq 0$ , for all p=1,2,…,P , are user-specified weights reflecting relative importance, $f_{p}$ are user-chosen individual objective functions, x is the beamlet radiation intensities vector (which is physically bound to the nonnegative real orthant), and $c_{t}$ are user-chosen individual constraints with lower and upper bounds $c_{t}^{L}$ and $c_{t}^{U}$ , respectively. While the constraints $c_{t}$ can, in principle, be nonlinear constraints, we focus here on *linear inequality constraints* representing upper and lower dose prescription bounds.

The inverse planning problem from eq. (1), solved with numerical optimization techniques, is commonly used today across treatment modalities (among others 2, 3, 5, 40, 41). SQP or interior point methods with a (quasi-)Newton approach are often used to solve the resulting constrained optimization problems (1–7, 42). In this work, we focus on a quasi-Newton approach using first-order derivatives only, since the superiorization approach (as described further below in section 2.4) has so far only been investigated using gradient descent steps itself.

### 2.3 The feasibility-seeking approach

Since the bare feasibility-seeking approach is the backbone of the SM, it will be outlined below using the notation from sections 2.1 and 2.2. Prior work has already suggested the feasibility-seeking approach to address the treatment planning problem (see, e.g., 43, and references therein).

To solve the treatment planning problem with feasibility-seeking, dose prescriptions are modeled as a system of linear inequalities: In general, the dose in every voxel is constrained with a lower and upper bound. Feasibility-seeking now seeks a solution, i.e., a beamlet intensity vector fulfilling these prescriptions.

With d(x)=Ax , the beamlet radiation intensities vector x now has to be recovered from a system of linear inequalities of the form


(2) $$c_{i}^{L}\leq \sum_{j=1}^{m}a_{i}^{j}x_{j}\leq c_{i}^{U},\;i=1,2,\ldots ,n.$$

In principle, individual lower and upper bounds $c_{i}^{L}$ and $c_{i}^{U}$ can be chosen for each voxel i . Since prescriptions are usually grouped per VOI $S_{q}$ , the system can be rewritten as:


(3) $$\text{For all}\;q=1,2,\ldots ,Q:\;\ell_{q}\leq \sum_{j=1}^{m}a_{i}^{j}x_{j}\leq u_{q}\text{ for all }i\in S_{q},$$

with $\ell_{q}$ and $u_{q}$ representing the lower and upper dose bounds per VOI $S_{q}$ , respectively. Since it does not make sense to prescribe positive lower bounds to OARs, these are generally chosen to be equal to zero.

Geometrically, depending on which structure $S_{q}$ a voxel i belongs to, each physical dose constraint set $C_{i}$ in each voxel i=1,2,…,n, is a *hyperslab* (i.e., an intersection of two half-spaces) in the *m*-dimensional Euclidean vector space ${\mathbb{R}}^{m}$ .

Aiming at satisfaction of all physical dose constraints along with the nonnegativity constraints is, thus, the following (which is a special case of the *convex feasibility problem* see, e.g., 23):


(4) $$\text{Find an}\;{\text{x}}^{*}\in \text{W}:=\{\text{x}\in {\mathbb{R}}^{m}|\text{for all},\;q=1,2,\ldots ,Q,\;\ell_{q}\leq \sum_{j=1}^{m}a_{i}^{j}x_{j}\leq u_{q},\text{ for all }i\in S_{q},\text{and}\;\text{x}\geq 0\}$$

Such feasibility-seeking problems can typically be solved by a variety of efficient projection methods, whose main advantage, which makes them successful in real-world applications, is computational (see, e.g., 23, 44).

They commonly can handle very large-size problems of dimensions beyond which other, more sophisticated currently available, methods start to stutter or cease to be efficient. This is because the building blocks of a projection algorithm are the projections onto the given individual sets. These projections are actually easy to perform, particularly in linear cases such as hyperplanes, half-spaces, or hyperslabs.

For the purpose of this paper, we define such an iterative feasibility-seeking algorithm via an algorithmic operator 𝒜 : ℝ^*m*^ → ℝ^*m*^ ,


(5) $${\text{x}}^{0}\in {\mathbb{R}}^{m},\;{\text{x}}^{k+1}=A\left({\text{x}}^{k}\right),\;k=1,2,\ldots \;,$$

whose task is to (asymptotically) find a point in W .

The algorithmic structures of projection algorithms are sequential, simultaneous, or in-between, such as in the block-iterative projection (BIP) methods (see, e.g., 45, 46, and references therein) or in the more recent string-averaging projection (SAP) methods (see, e.g., 47, and references therein). An advantage of projection methods is that they work with the initial, raw data and do not require transformation of, or other operations on, the sets describing the problem.

For our prototype used here in conjunction with the SM, we rely on the well-established Agmon, Motzkin, and Schoenberg (AMS) relaxation method for linear inequalities (48, 49). Implemented sequentially and modified for handling the bounds x≥0 , it is outlined in **Algorithm 1**. We denote $\ell :={\left(\ell_{q}\right)}_{q=1}^{Q}$ and $u:={\left(u_{q}\right)}_{q=1}^{Q}$ .

Algorithm 1 **The AMS Sequential Relaxation Method’s algorithmic operator *A^AMS^*.**

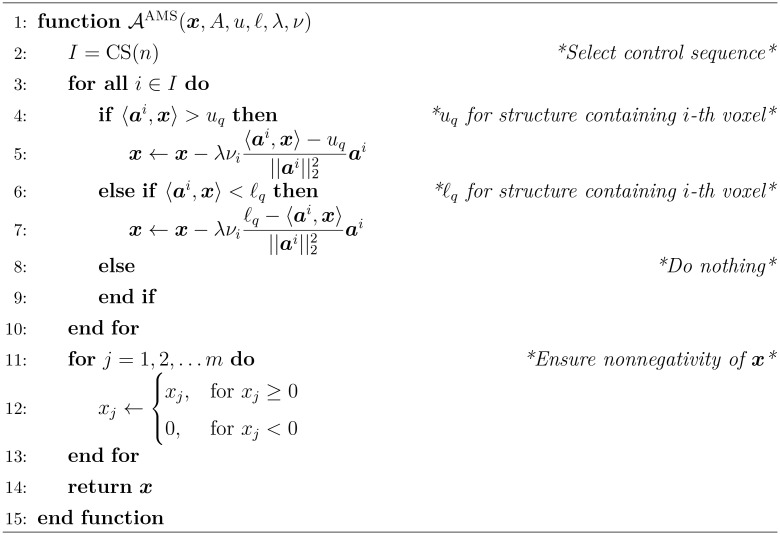

During an iteration, **Algorithm 1** iterates over all rows of the dose matrix *A* and handles sequentially the right-hand side and the left-hand side of individual constraints from eq. (3). The *control sequence* (CS) (50, Definition 5.1.1) determines the order of iterating through the matrix rows/constraints. When a corresponding voxel dose inequality is violated, the algorithm performs geometrically a projection of the current point x onto the violated half-space with a user-chosen relaxation parameter 0<λ≤2 . The original AMS algorithm is modified in **Algorithm 1** to allow the relaxation for each voxel i to be weighted with $\nu_{i}$ and by performing projections onto the nonnegative orthant of ${\mathbb{R}}^{m}$ (in steps 11–13) to return only nonnegative intensities x . The vector ${\text{a}}^{i}={\left(a_{i}^{j}\right)}_{j=1}^{m}$ is the i -th row of the dose matrix 𝒜 and is the normal vector to the half-space represented by that row and $\|{\text{a}}^{i}{\|}_{2}^{2}$ is its square Euclidean norm.

In summary, the algorithmic operator in **Algorithm 1** describes a single complete sweep of projections sequentially over all constraints (half-spaces) followed by a projection onto the nonnegative orthant thus ensuring the nonnegativity constraint. Such sweeps will be executed iteratively.

The theory behind this algorithm guarantees that, under reasonable conditions, if the feasibility-seeking sweeps are performed endlessly then any sequence of iteration vectors ${\left\{{\text{x}}^{k}\right\}}_{k=0}^{\infty }$ converges to a point that satisfies all constraints.

Choosing to define an algorithmic operator 𝒜 in **Algorithm 1**, allows us to concisely display the superiorization approach independent from the chosen projection algorithm below (see step 21 inside **Algorithm 2**).

### 2.4 The superiorization method and algorithm

The SM is built upon application of a feasibility-seeking approach (section 2.3) to the constraints in the second and third lines of eq. (1). But instead of handling the constrained minimization problem of eq. (1) with a full-fledged algorithm for constrained minimization, the SM interlaces into the feasibility-seeking iterative process (i.e., “the basic algorithm”) steps that reduce locally in each iteration the objective function value.

Accordingly, the SM does not aim at finding a constrained minimum of the combined objective function $f\left(\text{x}\right)=\sum_{p=1}^{P}w_{p}f_{p}\left(\text{x}\right)$ of eq. (1) over the constraints. It rather strives to find a feasible point that satisfies the constraints and has a reduced – not necessarily minimal – value of f .

In the following, we give a brief and focused introduction to SM. A more detailed explanation and review can be found in, e.g., Censor et al. (51, Section II) and references therein (see also 31, 35, 45, 52–55).

In general, the SM is intended for *constrained function reduction problems* of the following form (55, Problem 1):

**Problem 1. The constrained function reduction problem of the SM**

*Let* W *be a given set (such as in eq.* (4)*) and let* $f:{\mathbb{R}}^{m}\to \mathbb{R}$ *be an objective function (such as in eq.* (1)*). Let* 𝒜 *from eq.* (5) *be an algorithmic operator that defines an iterative basic algorithm for feasibility-seeking of a point in* W *. Find a vector* ${\text{x}}^{*}\in W$ *whose function value is smaller or equal (but not necessarily minimal) than that of a point in* W *that would have been reached by applying the basic algorithm alone.*

The SM approaches this question by investigating the *perturbation resilience* (52, Definitions 4 and 9) of 𝒜, and then proactively using such perturbations, to locally reduce the values f of the iterates, in order to steer the iterative sequence generated by algorithm A to a solution with smaller or equal objective function value. The structure of the superiorization algorithm implemented here is given by **Algorithm 2** with explanations here and in section 2.4.1.

Except for the initialization in steps 1–3, **Algorithm 2** consists of the perturbations phase (steps 5–19) and the feasibility-seeking phase (steps 20–23).

In the perturbation phase, the objective function f is reduced using negative gradient descent steps. The step-size β of these gradient updates is calculated by $\alpha^{s}$ where α is a fixed user-chosen constant, called *kernel*, 0<α<1 so that the resulting step-sizes are nonnegative and form a summable series. The power s is incremented by one until the objective function value of the newly acquired point is smaller or equal to the objective function value of the point with which the current perturbations phase was started.

The parameter N determines how many perturbations are executed before applying the next full sweep of the feasibility-seeking phase. The basic **Algorithm 1** with algorithmic operator 𝒜^AMS^, used throughout this work, is indeed perturbation resilient (56).

The superiorization approach has the advantage of letting the user choose any task-specific algorithmic operator 𝒜 that will be computationally efficient, independently of the perturbation phase, as long as perturbation resilience is preserved.

Algorithm 2 **Superiorization of the feasibility-seeking basic algorithm described by the operator *A = A^AMS^*.**

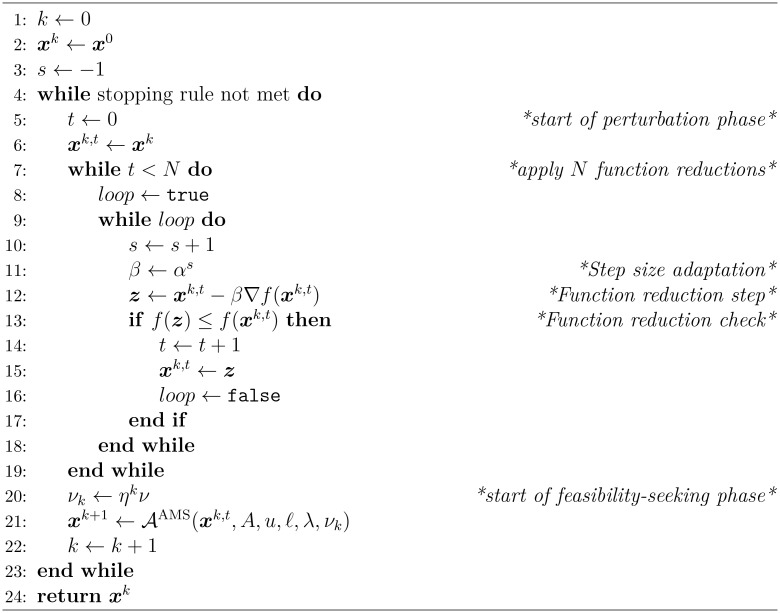

For our IMRT treatment planning problem using voxel dose constraints as introduced in eqs. (2) – (4), 𝒜 can be – besides the chosen AMS algorithm – any of the wide variety of feasibility-seeking algorithms (see, e.g., 23, 44, 50, 57).

The principles of the SM have been presented and studied in previous publications (consult, e.g. 31, 52, 54), but, to the best of our knowledge, this is the first work applying the SM to a treatment planning problem with an objective function of the general form $f\left(\text{x}\right):=\sum_{p=1}^{P}w_{p}f_{p}\left(\text{x}\right)$ from eq. (1).

#### 2.4.1 Modifications of the prototypical superiorization algorithm

To control the initial step-size, we warm start the algorithm with larger kernel powers s within the first iteration, which substantially improves the algorithm’s runtime. For our purposes, we chose an initial increment of *s* ← *s* + 25.

In the feasibility-seeking phase, instead of weighting all projections onto the half-spaces equally via the relaxation parameters, each projection can also be given an individual weight $0<\nu_{i}<1$ representing the importance of the i -th inequality constraint (i. e., voxel).

Further, as shown in step 20 of **Algorithm 2**, weights can be reduced after each iteration to improve stability. Similar to how the step-sizes are reduced in the perturbation phase, we utilize another kernel 0<η<1 and use its powers $\eta^{k}$ to reduce the weights in step 20 by incrementing k after each feasibility-seeking sweep. The new weights are then calculated by $\eta^{k}\cdot \nu ,$ where ν are the initial weights.

Finally, we integrate four different control sequences to iterate through the rows of A . Apart from following the cyclic order according to voxel indices, we experimented with a random order and with sequences choosing rows with increasing or decreasing weights $\nu_{i}$ .

#### 2.4.2 Stopping criteria

The algorithm was terminated after a given maximal number of iterations was reached or after a certain time limit was exceeded, or when the stopping criterion formulated below was met. The default number of maximum iterations was 500 and the default wall-clock duration was set to 50min.

The stopping criterion that we used consists of two parts, both of which must be met for three consecutive iterations for the algorithm to stop. The first part of the stopping criterion is that the relative change of the objective function f defined by


(6) $$\left|\frac{f\left({\text{x}}^{k+1}\right)-f\left({\text{x}}^{k}\right)|}{\text{max}\;\{1,f\left({\text{x}}^{k}\right)\}}\right|$$

becomes smaller than $10^{-4}$ .

For the second part of the stopping criterion, we define the square of the weighted $L_{2}$ -norm of the constraints violations by^2^


(7) $$V\left(\text{x}\right):=\frac{1}{n}\sum_{i=1}^{n}\frac{{\left(\ell_{q}-\langle {\text{a}}^{i},\text{x}\rangle \right)}_{+}^{2}+{\left(\langle {\text{a}}^{i},\text{x}\rangle -u_{q}\right)}_{+}^{2}}{\|{\text{a}}^{i}{\|}_{2}^{2}}$$

where $\ell_{q}$ and $u_{q}$ depend on which structure the i -th voxel belongs to. This second part of the stopping rule is met if the relative change of V defined by


(8) $$\left|\frac{V\left({\text{x}}^{k+1}\right)-V\left({\text{x}}^{k}\right)|}{\text{max}\;\{1,V\left({\text{x}}^{k}\right)\}}\right|$$

is smaller than $10^{-3}.$

All tolerances of the stopping criteria can be customized and also set to a negative number to turn off single stopping criteria or early stopping altogether.

### 2.5 Implementation

The superiorization prototype described above was implemented in the open-source crossplatform software “matRad” (5, 58, 59), which is a multi-modality radiation dose calculation and treatment planning toolkit written in Matlab. The implementation is publicly available on the matRad GitHub repository on a research branch.^3^

The superiorization solver is implemented as the class matRad_OptimizerSuperiorization.m within matRad's optimization framework. The class defines various user-configurable properties such as the maximum number of iterations, maximum wall time, different warm-start settings, two different feasibility-seeking algorithms, and various control sequences. Once the optimizer has been initialized, the optimize method can be called to generate a solution to the plan. The optimize method requires the following inputs: a starting point, the objective function with its gradient, the linear constraints, and the dose projection matrix. The perturbation phase, as well as the two provided feasibility-seeking algorithms, are implemented as additional methods. Furthermore, within the class, an additional method PlotFunction is available. This method facilitates the visualization of key metrics, such as the objective function value, the maximum constraint violation, and the proximity of the solution to the set of feasible solutions. Multiple scripts to reproduce the results presented herein are provided in an additional GitHub repository.^4^

The implementation in matRad facilitates comparison against plans generated on the same datasets with a nonlinear optimizer, as matRad implements a number of common objective functions used in treatment planning (compare to **Supplementary Data Sheet** and Wieser et al. (5, **Table 1**)). While matRad provides interfaces to both the open-source Interior Point OPTimizer (IPOPT) (19) as well as to Matlab’s built-in interior-point algorithm from fmincon, only the first was used for our comparisons.

We chose to use matRad’s optimization implementation as a benchmark for mainly two reasons: First, matRad has been used in numerous research works demonstrating its ability to create acceptable treatment plans. Second, as an open-source tool, matRad does allow direct modifications of the algorithms and respective parameters and stopping criteria, running them under truly similar conditions. This means that the evaluation of the objective function and its gradient itself use *exactly* the same code. Benchmarking against other closed-source treatment planning systems would be inconsequential due to hidden computational optimizations, simplifications, and unknown mathematical formulations of objectives and constraints.

As motivated in section 2.2, no second-order derivatives were used in the nonlinear optimization approach, but instead a limited-memory Hessian approximation using first-order derivatives was chosen. While second-order derivatives can be used within matRad, it does not make use of fast exact Hessian computation strategies (11), reducing the value of a runtime comparison.

matRad performs all computations in a fully-discretized model with a voxel grid. The “dose matrix” *A* is stored as a compressed sparse column matrix computed for all analyses using matRad’s singular value decomposed pencil-beam algorithm (60) for photons and a singleGaussian pencil-beam algorithm for protons, both validated against clinical implementations (5).

## 3 Results

### 3.1 Proof-of-work: Phantom plan

To demonstrate the applicability of superiorization to the IMRT treatment planning problem, we first evaluate a small example using the horseshoe phantom defined in the AAPM TG119 Report (61). The phantom is part of the CORT dataset (62) and consequently available with matRad.

We created an equidistantly spaced 5-field IMRT photon plan with 5mm *×* 5mm beamlet doses (resulting in 1918 pencil-beams and a corresponding sparse dose influence matrix with 9.3 *×* 10^7^ non-zero entries in 3.5 *×* 10^6^ voxels).

With this setup, we generated treatment plans using three different approaches: (i) constrained minimization with IPOPT, (ii) the AMS algorithm for feasibility-seeking only, and (iii) the SM with the AMS algorithm. Different combinations of nonlinear objective functions and linear inequality constraints on dose were evaluated and compared across these approaches.

For analysis, we use dose-volume histograms (DVHs) and axial dose (difference) slices, as well as the evolution plots of the objective function values and the constraint violations.

#### 3.1.1 General usability of the AMS feasibility-seeking projection algorithm

We first validate that our implemented projection algorithm AMS is capable of finding comparable treatment plans to those found by established optimization algorithms when applied to a straightforward piece-wise least-squares objective function for the unconstrained minimization of residuals.

The setup prescribes 60 Gy to the C-shaped target. To achieve this prescription, we bound the dose in the target by (60 ± 1) Gy. To the two OARs, “Core” and “Body”, upper bounds (a.k.a. tolerance doses) are prescribed, resulting in the parameters given in **Table 1**.

For nonlinear minimization with IPOPT, the tolerance doses serve as parameters for respective penalized piece-wise least-squares objective functions while for AMS the tolerances directly translate into linear inequalities and the weights proportionally increase the relaxation parameters.

**Figure 1:**
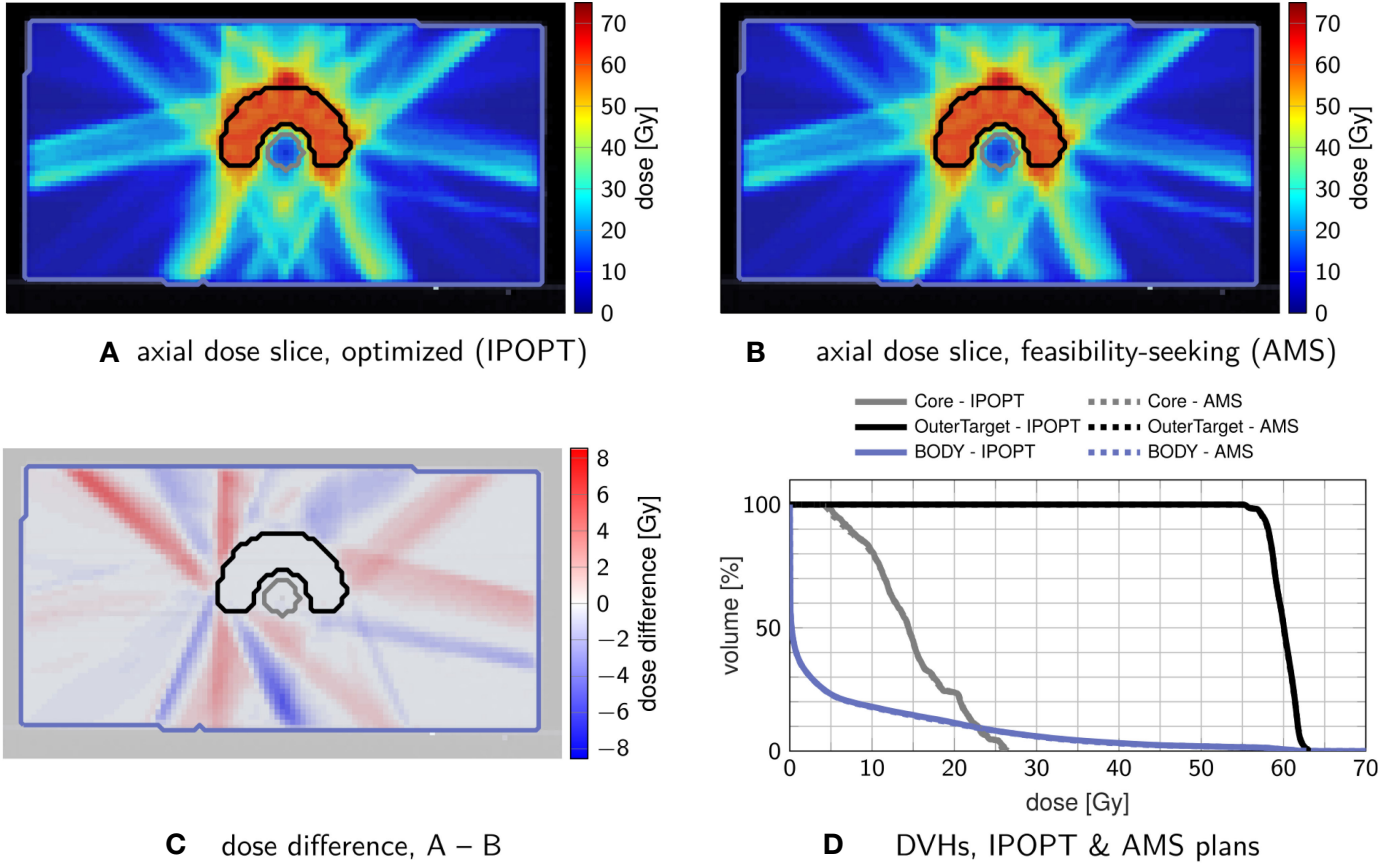
Comparison of treatment plans obtained by nonlinear minimization with IPOPT **(A)** and by feasibility-seeking with AMS **(B)**, using the tolerances from **Table 1**. **(C)** shows the dose difference in the slice from **(A, B, D)** the corresponding DVH, in which the optimization result (solid) and feasibility-seeking result (dashed) are nearly overlapping.

**Figure 1** confirms that feasibility-seeking with weighted AMS is able to find dose distributions of similar quality as conventional nonlinear unconstrained minimization of a piece-wise leastsquares objective function. While resulting in different intensity-modulation patterns, nearly congruent DVHs are observed.

A crude performance analysis though measures substantially longer runtimes for the AMS approach (about five times slower than unconstrained minimization). This difference is mainly driven by the fact that AMS does sequential iteration by iterations through the matrix rows in each sweep.

This investigated scenario is, however, not intended to display any performance advantages of the AMS algorithm, but only to validate its behavior and confirm the long-known ability of such feasibility-seeking algorithms to yield acceptable treatment plans (21, 22).

#### 3.1.2 Inverse planning with superiorization

Using the same phantom and irradiation geometry as in section 3.1.1, the feasibility problem used in 3.1.1 was modified to enforce some hard linear inequality constraints while minimizing an objective function. When the constraints are feasible, superiorization using AMS as the basic algorithm will find a feasible point while perturbing the iterates of the feasibility-seeking algorithm towards smaller or equal (not necessarily minimal) function values with objective function reduction steps.

As reference, nonlinear constrained minimization with IPOPT with a logistic maximum approximation for minimum/maximum (compare (5), **Table 1**), was used. Three prescription scenarios were investigated: (I) linear inequalities on the target (59 Gy<d<61 Gy) , (II) additional linear inequalities on the “Core” structure (d<30 Gy) , and (III) only linear inequalities on the “Core” (d<30 Gy) . The parameters are detailed in **Table 2**.

**Table 2:** Dose inequality constraints, objective functions, and penalty weights used separately for constrained minimization and for superiorization.

VOI	*w_p_*	*c*(*d*)	*f*(*d*)
Target	1000	59 Gy < ***d*** < 61 Gy (I & II)	*f*_sqdev_ (***d***; 60 Gy)
Core	100	***d*** < 30 Gy (II & III)	*f*_sqdev+_ (***d***; 20 Gy)
Body	30	–	*f*_sqdev+_ (***d***; 30 Gy)

The Roman numerals in parentheses for the inequality constraints describe their usage in the plans, respectively. The functions in the right-hand column stem from Wieser et al. (5, **Table 1**) and are identified here in **Supplementary Data Sheet** below.

**Figure 2:**
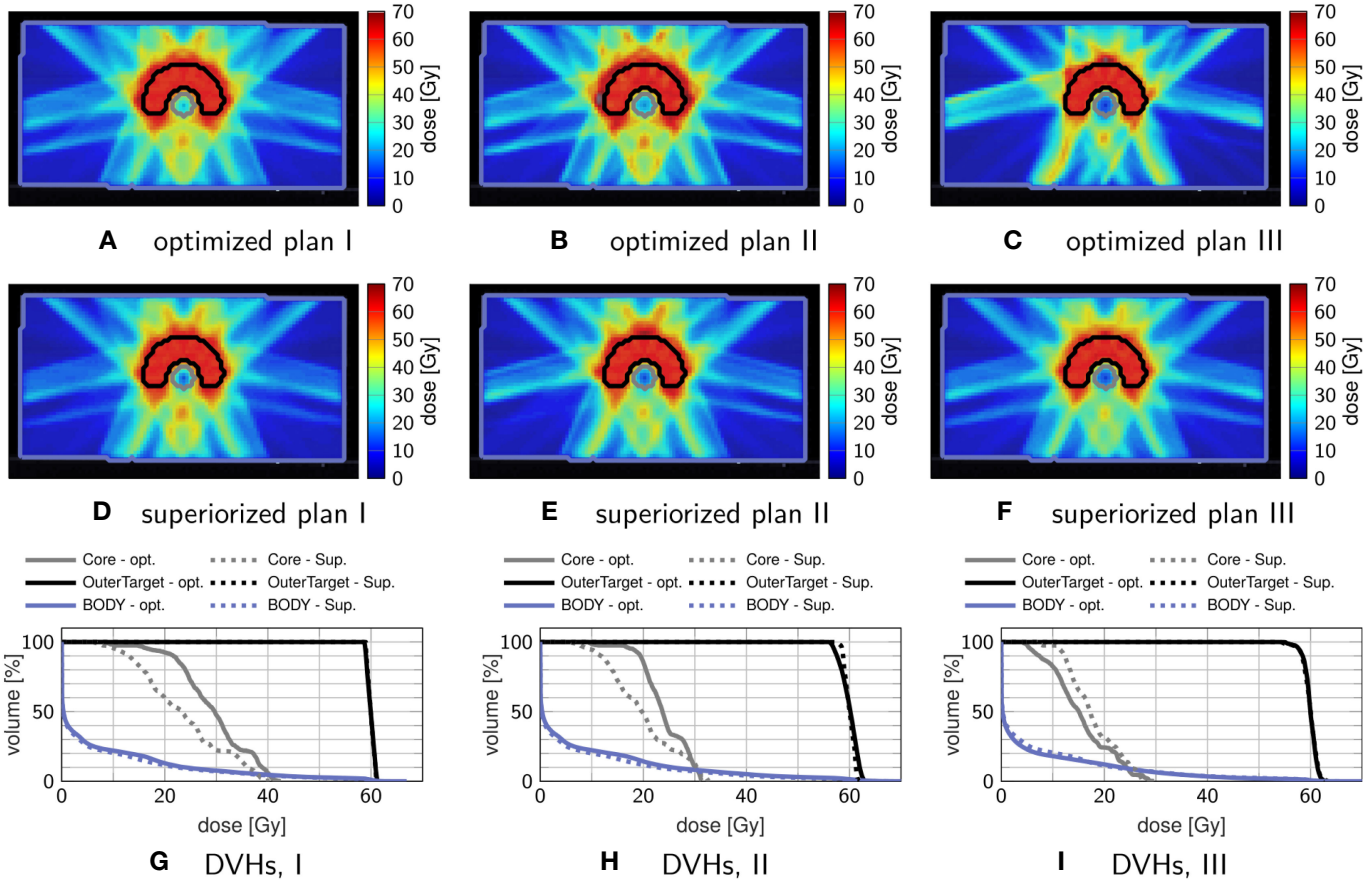
Comparison of treatment plans obtained by superiorization and by constrained minimization. The top row **(A–C)** shows axial dose distribution slices after constrained minimization, and the middle row **(D–F)** shows axial dose distribution slices after superiorization. The corresponding DVHs are shown in the bottom row **(G–I)**, with dashed lines showing the superiorization result and solid lines showing the optimization result.

**Figure 3:**
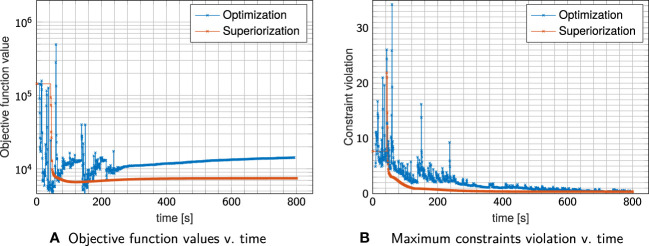
Objective Function values **(A)** and maximum constraint violation **(B)** over time for plan I shown in **Figure 2**. Each cross indicates a full iteration.

**Figure 2** compares dose distributions and DVHs after superiorization and after constrained minimization. The respective evolution of the objective function values and the constraint violations (calculated by the infinity norm over all inequality constraint functions, corresponding to the maximum residual) is exemplarily shown in **Figure 3** for plan I.

Comparing plan quality, both plans adhere to the linear inequality constraints when the problem is feasible (which is the case for plans I & III) as seen in the DVHs. In plan I, superiorization appears to reach better OAR sparing with reduced mean and maximum dose, while in plan III constrained minimization achieves better OAR sparing. For plan II, which poses an infeasible problem, both target coverage and mean OAR sparing are improved for superiorization, yet at higher OAR maximum dose than obtained through constrained minimization.

The evolution of the objective function and constraint violation for plan I in **Figure 3** exhibits a “typical” behavior of superiorization, seeing a strong decrease in the objective function values within the first iterations, followed by a slower slight increase as the perturbations’ step-sizes diminish. Both approaches were stopped after the maximum number of iterations (1000) was reached.

Nearly similar constraint violation is achieved by both methods, while constrained minimization resulted in higher objective function values than superiorization, which can be attributed to the difference in OAR sparing. For all investigated plans I–III, superiorization showed a much “smoother” evolution of objective function and constraint violation than observed in the constrained minimization approach.

### 3.2 Head-and-neck case

To prove the usability of superiorization in a conventional planning setting, we applied the SM to a head-and-neck case with a wider range of available objective functions, i.e., including common DVH-based objectives.

Coverage of the planning target volumes (PTVs) was enforced using voxel inequality constraints. Again, the results of superiorization were compared to those obtained by solving the constrained minimization problem. All objectives and constraints are given in **Table 3**.

**Table 3:** Dose inequality constraints, objective functions and penalty weights used for optimization and for superiorization on the head-and-neck case.

VOI	*w_p_*	*c*(*d*)	*f*(*d*)
PTV70	1000	66.5 Gy < ***d <*** 77 Gy	*f*_sqdev_(***d***; 70 Gy)
PTV63	1000		*f*_sqdev_(***d***; 63 Gy)
PTV63	1000		*f*_minDVH_(***d***; 60 Gy, 95%)
Spinal Cord PRV	100	***d <*** 50 Gy	*f*_sqdev+_(***d***; 15 Gy)
Parotid L & R	100		*f*_sqdev+_(***d***; 10 Gy)
Optic Nerve L & R	100		*f*_maxDVH_(***d***; 50 Gy, 10%)
Larynx	300		*f*_sqdev+_(***d***; 15 Gy)
Chiasm	100		*f*_maxDVH_(***d***; 50 Gy, 10%)
Cerebellum	100		*f*_sqdev+_(***d***; 15 Gy)
Brainstem PRV	100	***d <*** 30 Gy	*f*_sqdev+_(***d***; 15 Gy)
NT/Body	100		*f*_mean_(***d***)

The functions in the right-hand side column are identified here in **Supplementary Data Sheet**.

Both solvers use the same stopping criteria for the maximum constraint violation (smaller than 0.01 Gy is acceptable) and objective function change of value (smaller than 0.1% in three consecutive iterations/sweeps).

**Figure 4:**
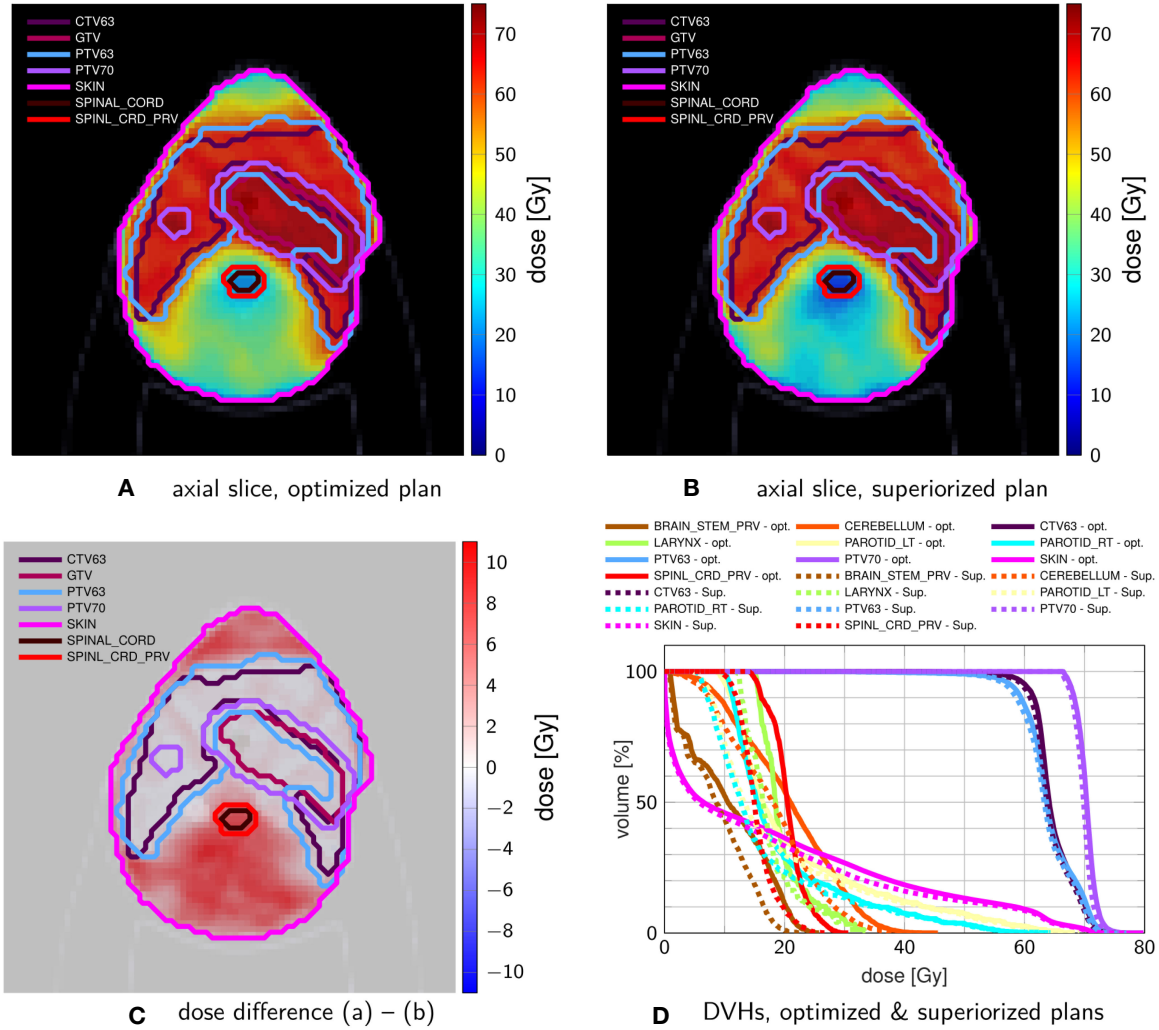
Comparison of head-and-neck treatment plans after **(A)** constrained minimization and after **(B)** superiorization (with AMS as the basic algorithm) using the tolerances from **Table 3**. **(C)** shows the dose difference in the same slice displayed in **(A, B)**. **(D)** compares the resulting DVHs after optimization (solid) and superiorization (dashed).

**Figure 5:**
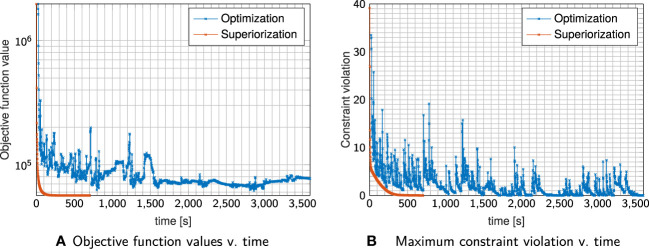
Evolution of objective Function values **(A)** and constraint violation **(B)** with runtime for the plan shown in **Figure 4**.

**Figure 4** shows exemplary axial dose slices and the DVHs for the plans generated with constraint minimization and with the SM. Quantitative runtime information and evolution of objective function and constraint violation are provided in **Figure 5**.

Both techniques were able to generate a plan that satisfies the linear inequalities up to the allowed violation threshold. Considering absolute runtime, the plan generated with the SM satisfied the stopping criteria after 400s, with constrained minimization failing to converge until the maximum number of iterations was reached.

SM spent most of the time in the first sweep/iteration, where it focuses on multiple objective function evaluations to generate a large initial decrease (as already observed above). It continuously decreases the objective function values together with decreasing constraints violation, reaching acceptable constraints violation more slowly than the run with constrained minimization.

However, using the same stopping criteria, the SM reached a solution with a much lower objective function value (approximately one-third of the value achieved by the constrained minimization plan). This is also visible in the dose slices and DVH, which show more normal tissue/OAR sparing for the SM plan. All results are, naturally, only valid for the experiments we performed. Further work, with varying algorithmic parameters, initialization points, and stopping criteria, is necessary to make more general statements.

### 3.3 Prostate case

To demonstrate how the superiorization approach translates to a second patient, using a different irradiation modality, we create prostate IMPT plans with opposing fields on a 5mm spot grid using both superiorization and constrained minimization.

**Figure 6:**
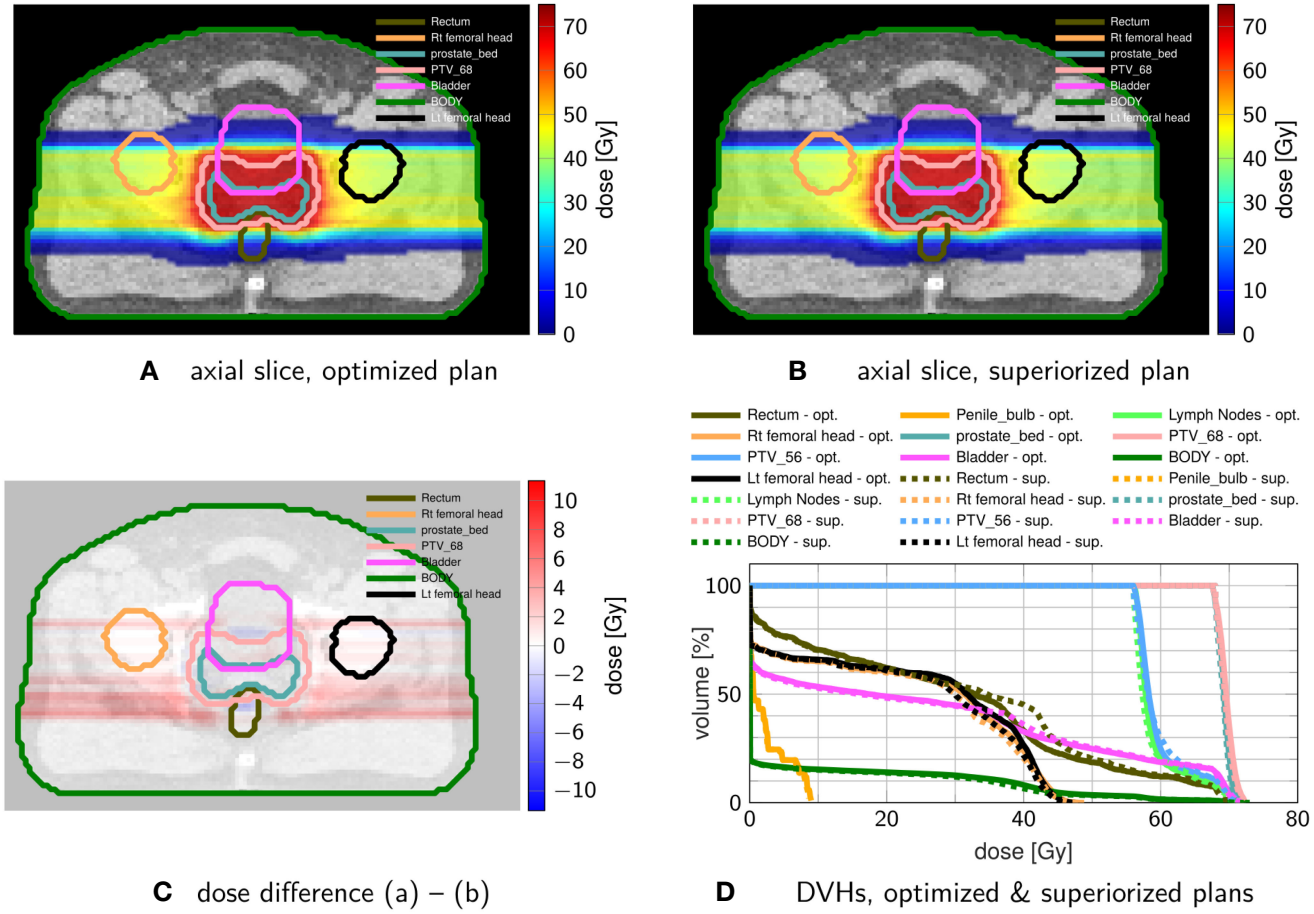
Comparison of prostate proton treatment plans after **(A)** constrained minimization with IPOPT and after **(B)** superiorization (with AMS as the basic algorithm) using the tolerances from **Table 4**. **(C)** shows the dose difference in the same slice displayed in **(A, B)**. **(D)** compares the resulting DVHs after optimization (solid) and superiorization (dashed).

**Figure 6** shows exemplary axial dose slices and the DVHs for the plans generated with constraint minimization and with the SM for the objective and constraint functions stated in **Table 4**.

**Table 4:** Dose inequality constraints, objective functions and penalty weights used for optimization and for superiorization on the prostate proton case.

VOI	*w_p_*	*c*(*d*)	*f*(*d*)
PTV68	5000	68 Gy < ***d <*** 72 Gy	*f*_sqdev_(***d***; 68 Gy)
PTV56	3000	56 Gy < ***d <*** 72 Gy	*f*_sqdev_(***d***; 56 Gy)
Rectum	100		*f*_sqdev+_(***d***; 30 Gy)
Rectum	300		*f*_maxDVH_(***d***; 50 Gy, 20%)
Bladder	100		*f*_sqdev+_(***d***; 30 Gy)
Bladder	300		*f*_maxDVH_(***d***; 50 Gy, 20%)
Femoral Heads	100		*f*_sqdev+_(***d***; 30 Gy)
NT/Body	100		*f*_mean_(***d***)

The functions in the right-hand side column are identified here in **Supplementary Data Sheet**.

The superiorized plan matches the dosimetric performance of the constrained minimization approach. Little increased dose in the rectum and bladder are traded against a slightly more homogeneous target coverage and reduced dose in the femoral heads.

## 4 Discussion

In this work, we applied the novel superiorization method, which solves a system of linear inequalities while reducing a nonlinear objective function, to inverse radiotherapy treatment planning. On a phantom and on a head-and-neck case, we demonstrated that superiorization can produce treatment plans of similar quality to plans generated with constrained minimization.

Superiorization showed a smooth convergence behavior for both objective function reduction and constraint violation decrease, including the “typical” behavior of strong initial objective function reduction with subsequent diminishing objective function reduction – including potential slight increase – while proximity to the feasible set within the dose inequality constraints is achieved.

### 4.1 The mathematical framework of constrained minimization and of superiorization for treatment planning

At the heart of the superiorization algorithm lies a feasibility-seeking algorithm (in this work, the AMS relaxation method for linear inequalities). This means that superiorization handles the treatment planning problem as a feasibility-seeking problem for linear inequality dose constraints that should be fulfilled while reducing (not necessarily minimizing) an objective function along the way.

Constrained optimization algorithms, on the other hand, tackle the same data, i.e., constraints and objective function, as a full-fledged optimization problem. With the IPOPT package, for example, inequality constraints become logarithmic barrier functions and are incorporated as a linear combination into the Lagrangian function, whose minimization then enforces the constraints (19).

When the problem is hardly feasible, finding the right Lagrange multipliers may then dominate the optimization problem in its initial stages. Superiorization with a feasibility-seeking projection algorithm will smoothly reduce the proximity to the constraints, even for infeasible constrained problems, while the perturbations in the objective function reduction phase reduce the objective function value.

Our current implementation is, however, specifically geared for linear constraints. Yet other works on feasibility-seeking have shown that other relevant constraints, like, e.g., DVH constraints, can be incorporated into the feasibility-seeking framework, since they can still be interpreted as linear inequalities on a subset (relative volume) of voxels (25–27)**.**

### 4.2 Comparability of runtime, convergence and stopping criteria

We demonstrated that feasibility-seeking for inverse IMRT treatment planning is practically equivalent to the least-squares approach if similar prescriptions are set. However, obtaining the final solution with feasibility-seeking took more time than with unconstrained minimization with our prototype implementation in Matlab.

Stopping criteria, convergence and runtimes are more comparable when considering the constrained minimization vis-à-vis superiorization. Our prototype superiorization algorithm “converged” as fast as the used constrained nonlinear minimization algorithm when using the same objective functions and linear inequalities, exhibiting smoother progress during the iterations. It is interesting to note that even so SM is not guaranteed to find an optimal solution it sometimes exhibits better initial behavior than the constrained minimization algorithm. A similar phenomenon has been observed in the past by Censor et al. (53), wherein the SM was compared with a projected subgradient method (PSM) on a CT image reconstruction problem from projections in computerized tomography.

Recognizing the limited scope of the experiments presented here, our results about the superiorization method need further work to become well established. For example, the stopping criteria play a substantial role in both optimization and superiorization. Further modification of the respective parameters may lead to earlier or later stopping of either of the algorithms. Particularly the quasi-Newton algorithm will likely improve on its solution when allowing more iterations/longer runtimes. However, we suspect that the Lagrangian is particularly difficult to navigate when using a Hessian approximation over exact Hessian computations in these heavily constrained examples. This suspicion is supported by a solver benchmark performed by ten Eikelder et al. (63).

Consequently, runtime and convergence of a constrained nonlinear optimization algorithm would expectedly improve when incorporating second derivatives, such as proposed by van Haveren and Breedveld (11), instead of relying on a low-memory approximation to the quasi-Newton approach. In addition, alternative nonlinear minimum/maximum dose constraint implementations are possible. An advantage of the SM is that such “workarounds” are not necessary.

For superiorization, computational complexity and convergence are heavily dependent on the chosen feasibility-seeking algorithm. While the function reduction in superiorization has the computational complexity of gradient descent steps, the basic AMS algorithm used as a starting point performs sequential projections over all constraints. The complexity is thus principally comparable to the corresponding submatrix-vector products, however, the algorithm’s sequential structure complicates parallelization and other computational optimizations. Thus, modifications of the AMS algorithm are still actively researched (e.g., 64). Computational complexity and convergence properties of projection algorithms are a topic of ongoing research (see, e.g., 65, where it is discussed in a more general setting).

Despite these limitations, we demonstrated that a straightforward superiorization implementation was able to solve the given treatment planning problem arriving at dosimetrically comparable treatment plans.

### 4.3 Dosimetric performance

The treatment plans obtained with constrained minimization and with superiorization show some dosimetric differences. For the three different linearly constrained setups on the TG199 phantom, these differences were most pronounced on the OAR, and less pronounced for the target dosage.

In the setups with target dose inequality constraints, superiorization reached better OAR sparing. This may be a result of multiple interacting factors: the strong initial objective function decrease in superiorization pulling down the dose in the OAR, and potential too early stopping of the constrained minimizer.

Further, in the infeasible setting with linear inequality constraints on both target and OAR, superiorization has the advantage that the feasibility-seeking algorithm will still smoothly converge to a proximal point.

The improved OAR sparing did not occur when only using dose inequality constraints on the OAR. However, in this case, the differences in DVHs of the OAR are only substantial below a dose of 20Gy and, thus, of limited significance, since a piece-wise least-squares objective was used that does not contribute to the objective function at dose values below 20Gy.

The head-and-neck case also reproduces the better OAR sparing for all evaluated OARs, at slightly reduced target coverage for the non-constrained CTV63 and PTV63. Here, the difference in convergence speed was most significant. Through all cases, the superiorization exhibited the smooth evolution of both objective function value and constraint violation, which in turn suggests robustness against changes in the stopping criteria as well. This behavior of superiorization could be underlined by translating it to IMPT on a prostate case.

These encouraging results show that superiorization can create acceptable and apparently “better” treatment plans. Additional work on more cases or planning benchmarks, with varying tuning parameters of both constrained minimization and superiorization approaches is needed to assess the convergence, runtime, and dosimetric quality of the solutions.

### 4.4 Outlook

With the proof-of-concept put forward in this work, there are many possible directions to further investigate the application of superiorization algorithms to the radiotherapy inverse treatment planning problem. From the perspective of a treatment planner, one may focus on enabling further constraints, e.g., DVH-based constraints, that are often used in treatment planning.

Some of these constraints are also representable as modified linear inequalities or convex and non-convex sets and, thus, can efficiently be solved using a feasibility-seeking algorithm. Even nonlinear constraints that are based, for example, on normal-tissue complication probability or equivalent uniform dose could be incorporated in the current definition of the superiorization algorithm if the “basic algorithm” in the feasibility-seeking phase of the SM is replaced by any other perturbation resilient projection method that can handle nonlinear constraints. Such algorithms exist in the literature.

Moreover, superiorization might also be extended to use more complex function reduction steps and inherent criteria. For example, a “true” backtracking line search could be performed, similar to approaches in optimization, since a perturbation resilient “basic algorithm” might be able to handle much more complex function reduction steps.

Considering these algorithmic and application-focused improvements, the SM should also be rigorously tested on radiotherapy optimization/inverse planning benchmark problems, like the TROTS dataset (66), as soon as it is able to handle the respective problem formulations. With this, transferability to other modalities like ion therapy or volumetric modulated arc therapy (VMAT) is also within reach.

## 5 Conclusions

We introduced superiorization as a novel inverse planning technique, merging feasibility-seeking for linear inequality dose constraints with objective function reduction. Our initial comparison of superiorization with constrained minimization using linear dose-inequalities suggests possible dosimetric benefits and smoother convergence. Superiorization is thus a valuable addition to the algorithmic inverse treatment planning toolbox.

## Data availability statement

All data used in this study is publicly available through matRad. The code to reproduce our results is available on GitHub: https://github.com/e0404/paper-superiorization-imrt. Further inquiries can be directed to the corresponding authors.

## Ethics statement

Ethical approval was not required for the study involving humans in accordance with the local legislation and institutional requirements. Written informed consent to participate in this study was not required from the participants or the participants’ legal guardians/next of kin in accordance with the national legislation and the institutional requirements.

## Author contributions

NW and YC conceived the original idea for the research. YC developed the theoretical framework for the study, while FB implemented the method. NW conducted the analyses of the algorithm. All authors contributed to the writing and editing of the manuscript. All authors contributed to the article and approved the submitted version.

## Footnotes

1 Censor (http://arxiv.org/abs/1506.04219 ) provides a continuously updated bibliography of works using superiorization to present the state of research (30).

2 For any real number *r* we use (*r*)_+_ := max {0, *r*}

3
https://github.com/e0404/matRad/tree/research/superiorization. Commit hash at the time of this work: ec04f5a5

4
https://github.com/e0404/paper-superiorization-imrt/. Commit hash at the time of this work: a787e94 .
