# Supplementary material for: Superiorization of projection algorithms for linearly constrained inverse radiotherapy treatment planning
Source: Front Oncol. 2023 Oct 26;13:1238824. doi: 10.3389/fonc.2023.1238824 (PMC10685292; doi:10.3389/fonc.2023.1238824)
Supplement: Supplementary file 1 [file DataSheet_1.pdf]

Supplementary Material – Objective Functions

The nonlinear objective functions used in this work correspond to the implementation in matRad[a]:

$$
\begin{aligned}
f_{\text{sqdev}}(\boldsymbol{d};\hat{d}) &= \frac{1}{n}\sum_i (d_i - \hat{d})^2 \\
f_{\text{sqdev+}}(\boldsymbol{d};\hat{d}) &= \frac{1}{n}\sum_i \Theta(d_i - \hat{d})(d_i - \hat{d})^2 \\
f_{\text{sqdev}-}(\boldsymbol{d};\hat{d}) &= \frac{1}{n}\sum_i \Theta(\hat{d} - d_i)(d_i - \hat{d})^2 \\
f_{\text{minDVH}}(\boldsymbol{d};\hat{d},v) &= \frac{1}{n}\sum_i \Theta(\hat{d} - d_i)\Theta(d_i - D_v(\boldsymbol{d}))(d_i - \hat{d})^2 \\
f_{\text{maxDVH}}(\boldsymbol{d};\hat{d},v) &= \frac{1}{n}\sum_i \Theta(d_i - \hat{d})\Theta(D_v(\boldsymbol{d}) - d_i)(d_i - \hat{d})^2 \\
f_{\text{mean}}(\boldsymbol{d}) &= \frac{1}{n}\sum_i d_i
\end{aligned}
$$

All objective functions are evaluated on a dose vector $\boldsymbol{d}$ of length n, which corresponds to the number of voxels in each volume of interest. $D_v(\boldsymbol{d})$ is the dose at least received by the volume fraction v and thus corresponds to the respective point in the dose-volume histogram. The parameter $\hat{d}$ represents a prescribed/tolerance dose.

---

[a] Wieser HP, Cisternas E, Wahl N, Ulrich S, Stadler A, Mescher H, et al. Development of the open-source dose calculation and optimization toolkit matRad. Medical Physics 2017;44:2556–68, Table 1.
